# How i read cancer imaging studies: the master class series

**DOI:** 10.1186/s40644-016-0067-3

**Published:** 2016-04-11

**Authors:** Rodney J. Hicks

**Affiliations:** Cancer Imaging, The Peter MacCallum Cancer Centre, St Andrew’s Place, East Melbourne, VIC 3002 Australia; The Sir Peter MacCallum Department of Oncology, The University of Melbourne, Parkville, Australia

## Abstract

The generation of a report of findings and a conclusion for an imaging study is a fundamental process in the diagnostic work-up of cancer patients and plays a critical role in treatment decisions. It is therefore important that cancer imaging specialists understand the needs of referring clinicians and can perform this process in a methodical manner. Surprisingly, in contrast to the literature regarding the outcomes of imaging, education regarding the methods that underpin the generation of a report and how to communicate the findings cogently to referring clinicians is rather sparse. In an upcoming series of articles that will appear in in *Cancer Imaging*, experts in various modalities will detail their approach to reporting scans in particular disease settings. In this article some personal perspectives on the process of reporting scans will be detailed as a guide to what will be elaborated in the “Master Class Series”.

## Introduction

Working, as I do, in a busy cancer imaging department that is actively involved in training of fellows and visiting imaging specialists, I have become acutely aware of how many basic processes involved in displaying, reviewing and interpreting scans I take for granted. Preceding the images even arriving at the workstation for reporting, decisions have been made about patient preparation and study acquisition that influence the material available for review. Most large departments have established protocols to standardise these technical aspects of imaging, and default settings come preloaded by manufacturers of imaging equipment or DICOM viewing software. However, with all these steps successfully negotiated, specialist review of the acquired images is required for an informative report to be generated. Without specific teaching, trainees develop their own method of reviewing images and a personal style of reporting, often assimilating what they observe during reporting sessions with more senior members of their local team. Meanwhile, most formal teaching focuses on the identification and description of disease and ignores the procedure of reading scans and communicating the findings.

Similarly, while a great deal of literature exists about typical disease findings, often by way of atlas-based teaching, and regarding the accuracy or impact of imaging studies in clinical practice, there is relatively little guidance on those, seemingly, more mundane processes of image interpretation, including windowing of scans and the order in which image sets are reviewed. Often the technical aspects of scanning are highly abbreviated to meet word limits set by print journals or simply omitted as being irrelevant to the focus of the paper. Yet like a golfer who wants to improve his or her swing by focussing sequentially on the grip, the position of the feet, head alignment, backswing, point of contact and follow-through, dissecting the process of cancer imaging study acquisition, interpretation and reporting is vital to improving the end product. For us, an accurate study that succinctly answers the clinical question posed by the referring doctor is our hole-in-one.

What better way to dissect these processes than to ask an expert coach? With *Cancer Imaging* being the official journal of the International Cancer Imaging Society, we have the luxury of access to recognised leaders in the field of oncological imaging with expertise that spans many modalities and diseases. Our Section Editors reflect and co-opt this expertise in their daily work. These same experts frequently run Master Classes as part of the annual teaching course of the International Cancer Imaging Society or during satellite meetings. It, therefore, seemed to me that this invaluable experiential resource should be made available to our readership. Accordingly, I have commissioned our Editorial Board to either write themselves, or to invite respected colleagues to write, a series of articles that don’t reflect a traditional review of the literature pertaining to their areas of interest but rather to crystallise their own personal approach to reading scans in particular cancer settings. These will focus on how they display, manipulate, and sequence images to assemble the pertinent findings required to reach a conclusion to a clinical question. Since these reviews will necessarily reflect highly personal approaches, moulded by experience and their own influencers, they won’t necessarily be the same as other experts might recommend. However, they will hopefully provide someone entering the field a foundation on which they can build their own framework. We hope that this will act in print as a surrogate of a hands-on master class and stimulate mentors and teachers to discuss their own approach with their trainees.

## How I read cancer imaging studies

While I will leave it largely to my esteemed colleagues to write reviews on specific subjects pertinent to their areas of interest, I would like to share my own general approach to reporting of cancer imaging studies. My *modus operandi* has developed over the past quarter of a century and reflects the positive influences of many inspiring mentors but also lessons learnt from my own errors. I can assure you that there have been many opportunities for cancer to find me out. It is a harsh critic! Its often remorseless progression unmasks the subtle and not-so-subtle abnormality that one overlooks, prodding one always to read more sensitively until one is equally chastised by the false-positive result that has committed a patient to unnecessary intervention or treatment. I have learnt that more harm comes to patients from overcalling than ignoring subtle abnormalities [1].

Having trained first as a physician, my internal medicine background strongly influenced a problem-oriented approach. In my initial studies around the diagnostic process in cardiology, I was also highly influenced by Bayesian principles [2]. I think that these two factors have stood me, and I hope my patients, well when applied to the field of cancer imaging.

When I was a young doctor doing a country rotation, I was fortunate to work with a very wise and excellent family physician. He told me that there are three steps to being a good doctor. The first is to take a history from the patient until they have told you everything that they need to say. The second is to do an examination until you have found everything that you need to know. The third is to come up with a diagnosis that you can both live with.

I’ve tried to apply these same principles to reporting of scans. My first step is formulation of the clinical question being posed. It is somewhat depressing how seldom the precise reason for a request is actually articulated on the written request form. Divining what the question is can involve review of the patient’s clinical notes, questioning the patient directly, perhaps via a standardised questionnaire, or, in need, by calling the referring doctor. Another of my former mentors told me that the most useful diagnostic tool in radiology is the telephone. To formulate in your own mind a question to be addressed informs a systematic approach to the next phases of the reporting process but also requires a significant knowledge base. I sometimes think that the most important part of training a young imaging specialist to interpret oncological studies is not so much to recognise abnormalities but rather to understand oncological principles and the questions that are important to clinicians managing patients with cancer. These include an understanding of basic treatment paradigms in a given disease. In particular, when loco-regional therapies such as surgery or radiotherapy are appropriate, the need to exclude distant metastatic disease becomes pivotal. My first question to myself and to my fellows when looking at a scan is, “What is the question here?”

Next comes the examination. Just as we were taught as medical students, general inspection should precede more detailed and specific eliciting of signs of disease. As with clinical examination, optimal viewing requires good lighting. For imaging, this involves appropriate thresholding or windowing of images, which is a process that varies according to modality and its application. This process will be detailed in the specific articles in this series. In PET and general nuclear medicine we have long had the luxury of having an overview of the scan in the form of a maximum intensity projection (MIP) image, or of a whole body planar scan in the anterior and posterior projections. These images provide a gestalt of the sites of abnormality. Often within milliseconds of these images being viewed, a diagnosis can be made regarding the presence or absence of abnormality and, based on pattern recognition, of the likely diagnosis. Although MIP image generation is becoming more prevalent in anatomical imaging, it is generally more difficult to gain such a rapid overview of the whole body for CT and MRI. For tomographic imaging, the next thing that I do is to scroll through the coronal image set. Although tomographic imaging historically developed as an axial scanning technique [3], the body actually makes much more sense when viewed in the coronal plane as most tubular structures run vertically or transversely in this plane rather than being rounded. I will return to the significance of this later in discussion of ‘Rod’s Rules’. Any apparent abnormalities observed on scrolling through these images are then triangulated and viewed in the sagittal and transaxial planes. For PET/CT, I look first at the stand-alone PET images and then at the CT images, first on the soft tissue window, then on bone windows and finally on lung windows. For assessing the liver, further windowing to optimise lesion visualisation on non-contrast CT may be appropriate. Although I have noted a tendency for neophytes in hybrid imaging to devolve immediately to simply scrolling through the fused transaxial images and then correlating any abnormality found on the stand-alone CT image, I have found that my systematic approach provides greater sensitivity and specificity in identifying abnormalities and recognising normal variants. I actually see the fused images as a marketing tool! If an appropriate colour scale is utilised, the resulting fused images provide an intuitive representation of pathological findings that clinicians have readily embraced. Further discussion of the use of colour scales in imaging will be included in later articles in this series.

By the time I’ve done this evaluation, I have generally formulated in my mind how I am going to address the clinical question. For example, if the primary purpose of the scan is to stage a known malignancy, I adopt a format based on the TNM staging system. Focussing first on the primary tumour, the qualities of the lesion are described. For PET this involves a description of the intensity and homogeneity of uptake and pertinent localising details followed by the associated radiologic features of relevance, including size, outline, calcification, necrosis and relation to key anatomical landmarks that help to define T-stage. At this point, I will also mention any direct complications or confounding factors. These might include effusions, collapse or features of infection. Thereafter, nodal disease, if present, is described from proximal to distal stations along with the qualitative features and, if pertinent, quantitative parameters of intensity and size. The description of stage is completed by identifying any suspected distant metastases, grouped by organ system. As part of this description I mention, particularly, those that might be at risk of complications, such as cord compression, fracture or obstruction. I also indicate pertinent negative findings. For example, if a given cancer has a predilection to spread to a particular organ, for example, ocular melanoma to the liver or prostate cancer to bone, I indicate that these sites are not involved. This helps to reassure the referring clinician that I am both aware of metastatic patterns and that I have carefully assessed these sites. Further, abnormalities that might be confused as being metastases but have some characteristics that allow assignment of a benign aetiology are mentioned. In PET, lung granulomas and adrenal incidentalomas are probably the most common. Importantly, I detail any additional findings that might be relevant to the management of the patient. For example, the presence of vascular disease, particularly coronary artery calcification in a patient who might be being considered for major surgery or active inflammation in someone who would soon receive chemotherapy, since these can be a source of septicaemia during any period of neutropaenia. Finally, I describe any abnormalities or normal variants that are irrelevant to the question being addressed. I tell my fellows that at the end of this description, the reader should be able to imagine the scan without having the images available. We do, however, routinely insert representative save screens into our reports.

The routine is the same but the description differs if the question relates to therapeutic response, suspected residual disease or relapse. For these, reference is made to prior findings, when available with focus on the characteristics, location and associations of sites of residual abnormality. Again, there is attention to pertinent negative findings and abnormalities that are the result of prior treatment rather than the disease itself.

## Rod’s rules

A critical part of cancer imaging is the assessment of whether abnormalities reflect malignancy or are relevant to the management of the patient. Since benign lesions, non-oncological pathologies and even second malignancies can co-exist with cancer or develop following successful treatment of cancer, being able to provide the managing clinician with guidance on the likely nature of abnormalities can be extremely helpful.

Over the years I have developed certain principles that help me in this process and that I share with all our trainees. I call them Rod’s Rules.

### Rod’s rule number 1

*Cancers grow like cities; radially until they meet a physical barrier, which they spread along, or are given a road to follow.*

Thus, most cancers, if unrestrained grow into spheres and thus appear rounded in each orthogonal projection. If constrained by an anatomical boundary, like the pleura or muscularis layer of the gastrointestinal tract, they will spread along this boundary. In the gut, this sub-mucosal spread leads to tumours appearing fusiform. Once a tumour breaches a physical barrier, and enters a new space, it again adopts a radial growth pattern that leads to the presence of lobulation. Alternatively if it enters a structure where it can follow a line of low resistance, it does so. The most obvious paths are lymphatics, leading to spiculation, but others include the pleural and peritoneal spaces and bloods vessels, particularly veins, which are easy to tumours to invade than more muscular arteries. As tumours extend into spaces they become more plaque-like whereas within a vessel they become tubular. Inflammatory processes are less respectful of physical barriers and tend to rely on direct permeation or passage through tissue via pathogenic portals of entry.

### Rod’s rule number 2

*Just as children generally resemble their parents, metastases generally bear significant resemblance to the characteristics of the primary lesion and if they don’t, only genetic testing can confirm their lineage.*

Although heterogeneity is increasingly being recognised as a feature of the evolution of cancers, generally there are more similarities between the primary cancer and its metastases than there are between the cancer and normal tissues. This is reflected in their imaging phenotype. For example, mucinous primary tumours will generally give rise to mucinous metastases. On PET, the presence of an intensely FDG-avid primary significantly diminishes the likelihood that an enlarged adrenal without significant tracer uptake represents a metastasis. With the increasing use of hybrid anatomical and molecular imaging, discordance between the interpretation of abnormalities on one component of the scan and the other may need biopsy to determine the nature of the abnormality. Alternatively, different molecular imaging signals between lesions that might otherwise be interpreted as being part of the same process can reflect synchronous or metachronous primaries. Differentiating between a metastatic process and occurrence of a tumour that might still be treated curatively can have significant positive implications for patient care and is not nearly as uncommon as one might think [4].

### Rod’s rule number 3

*As security agencies have learnt, if members of a crowd are behaving differently from the rest of the crowd, they are probably not part of it and need to be watched. Presumed metastases that are not responding in parallel with other lesions to treatment might reflect a different disease.*

Mixed responses in cancer are relatively uncommon. Typically all deposits respond, or not, relatively consistently to treatment, either regressing or progressing over time. When a lesion behaves differently from the rest, it is likely to represent either a different disease process or a resistant clone of cells. Accordingly, characterising such lesions by biopsy or molecular imaging can provide insight into their nature. We commonly see transformed lymphoma responding rapidly and completely to immuno-chemotherapy, whereas more indolent clones of low-grade follicular lymphoma can shrug off this treatment, remaining enlarged on CT. Heterogeneity of response is more prevalent with targeted therapies. Cells lacking the target will remain effectively untreated and will, if it is their nature, grow despite dramatic responses in lesions that contain exclusively or dominantly cells that express the target of the therapeutic agent. Mixed responses are less common with conventional cytotoxic therapies and therefore differential responses more commonly represent a different pathological process.

When assessing therapeutic response, it is important is important to not only compare the current scan with that immediately preceding it, but also to review prior scans. Lack of response in a lesion that had been stable prior treatment increases the likelihood of a benign aetiology. Conversely, by providing a greater interval between scans, it is sometimes possible to appreciate more subtle progression and regression of disease than can be evaluated from two scans that are separated by only a few weeks or months.

## Formulating a conclusion to a cancer imaging report

The conclusion of an imaging report should not simply be a reiteration of the findings. The first and most important aspect of a conclusion is, in my opinion, to directly address the clinical question. This should also recognise the level of knowledge and interest of the recipient. While any medically trained person should easily understand the conclusion, it should also be tailored to the training of the recipient. For example, a report regarding prostate cancer findings might be different if being sent to a surgeon compared to a radiation oncologist. Ideally, the level of confidence regarding a given set of findings should be communicated rather than simply a list of differential diagnoses. When the results are truly equivocal, this should be accompanied by suggestions on how a diagnosis might be reached, especially if alternative imaging might help. If appropriate, given the relative oncological expertise of the referring clinician and the imaging specialist, management guidance could be given. In areas of oncology in which I have both diagnostic and therapeutic experience, I am quite comfortable recommending treatment options for patients on the basis of scan findings. Working in a multidisciplinary environment, as is increasingly common in oncology, the report should address issues that might be pertinent to a number of involved craft groups.

As well as detailing positive findings that justify the conclusion, any highly pertinent negative findings should also be articulated. Finally, incidental findings that may warrant further investigation that might influence further management choices, should be detailed.

## A final word

I hope that you are looking forward as much as I am to the guidance of our Editorial Board and their esteemed colleagues on how to read scans and formulate a report in their areas of specific expertise.
